# The New World Language

**Published:** 1888-06

**Authors:** Frederick Whittaker


### THE NEW-WORLD LANGUAGE.

Many attempts have been made since facilities of transport made the modern man a world traveller, to provide for his use a language which would carry him everywhere with the least possible trouble. Five hundred years ago travellers were a wonder, language an impassible barrier to intercommunication between all but the learned and opulent: to-day, travellers are the rule; stay-at-homes the exception.

But in exact proportion to the increase of travel has come the need of some means of communication of ideas that will enable a traveller to go from country to country without being at the mercy of the professional "interpreter," "cicerone," "dragoman," "commissionaire" or "courier," who, at present, confronts him at every foreign port, side by side with the custom house officer. To be in a foreign land with nothing but your own language to fall back on, means to be subject to imposition at every turn, and to be compelled to pay for experience at every store and counter. Americans, who, as a rule, know no language but their own, fail to realize this fact till they get rich enough to take the "European trip;" but Europeans who have to face a fresh nation every time they travel between places no further apart than New York and Cincinnati, have the need of a universal language forced on them in a manner we can hardly appreciate in America. On this continent we have, practically, but two languages to master, with a third closely allied, if we travel from Labrador to Cape Horn, English, Spanish and Portugese will take us all through. French, while valuable in Montreal, is not an absolute necessity ; English being spoken in Canada as freely as French ; but Spanish is indispensable to any one who crosses the Rio Grande, while Portugese is equally a necessity to the man who goes to Brazil.

Luckily for American trade, Spanish is one of the easiest of modern languages to acquire ; but that very fact renders Americans more indolent than they might otherwise be, in a linguistic point of view. They are apt to consider time spent on the study of foreign tongues, wasted, principally, on the ground that they will never need any save Spanish, which can be acquired in a few months. Such, at least, used to be universally the case. Times are changing fast since ocean travel has increased and ocean cables multiplied.

In Europe, however, attempts have been made, for the last two centuries, to construct some language which might come into universal use, beginning with one made by Professor Leibnitz, in 1700. Leibnitz was one of the greatest logicians, mathematicians and philologists of his day, inventor of the calculus, metaphysician and thinker of the highest order ; yet his system failed to gain acceptance, simply because the time was not ripe therefor. Since his day, those who are curious on the subject have unearthed no less than fifty different attempts to construct such an universal language, all emanating from men of the highest capacity and intelligence ; but all failing to gain acceptance, from various causes.

Yet, the need of such a language becoming greater and greater, daily, the attempts contined, till, in 1879, a certain Roman Catholic parish priest living at Constance, in the duchy of Baden, by the borders of Lake Constance, published an outline of his proposed international language.

Up to that time this priest had been wholly unknown. He had no mathematical fame, like Leibnitz ; no literary renown, like Walter Savage Landor, to give distinction to his efforts, but where *they failed* to invent a practical language, *he succeeded.* He had the happiness within nine years from the time that he gave to the world his first outlines of what he called "Vol-a-pük" or "World's speech," to see it accepted into practical commercial use, and to know that grammars of the new language had been published in (quoting from the latest bookseller's catalogue) the following tongues : Basque, Chinese, Danish, Esthnish, Dutch, French,

Italian, Spanish, German, Portugese, Servian Croat, Turkish, Modern Greek, Swedish, Slavonian and Hungarian.

The name of this priest who sprung into fame with such a sudden bound, is Johann Martin Schleyer, and he is now fifty-seven years old. Preparatory education, by the acquirement of fifty distinct languages, was the method taken by him to select the elements that would fuse most easily. From all, he took what he wanted, with such success that Volapük became what no "universal language," up to the date of its promulgation, had become, a *practical tongue.*

It is not generally known, but it is nevertheless the fact, that some hundred and fifty thousand people in Europe alone, have already mastered Volapük, while thirteen periodicals devote a portion of their space to a Volapük department. Four more, published solely in the language itself, divide the suffrages of learners, according to the catalogues of New York booksellers.

A list of these may prove interesting to the readers of the JOURNAL OF HEALTH: First, take the "Kaled Volapükelas," or "Calendar of Volapükists," published in Munich, for twenty-five cents. Follow with the "Cogabled Volapükelas," or Joke paper" of the same, a comic journal in twelve numbers, $1.35 per year. Porto Rico has the "Timabled Volapükik," or "Time paper," (Anglice "Journal") of Volapük. Paris has "Le Volapük;" Madrid, "El Volapük;" Constance issues the Volapükabled Zenodik" or "World speech paper Central," edited by Schleyer; while Leipsic, Vienna and Breslau issue periodicals, almanacs and monthlies.

Facts of this sort show that the new language is no longer a theory, but a living reality. The experience of those who advocate Volapük is that the publication of a good grammar and dictionary of the new language in any tongue, is the sure signal for a wave of success, which increases momentarily. In England, there is no such book; in America there was none, this time last year; but since last November, the difficulty has been removed by a publication emanating from an American in New York City. Sprague's "Handbook of Volapük," has filled the gap in America, and has been the means, already, of starting large and enthusiastic "Volapükaklubs."

Having said this much concerning the success of the new language, the question remains for the ordinary reader: what causes have produced this effect and what kind of a language is it?

The answer to these queries is about as follows:

Volapük has succeeded on account of its marvellous simplicity, its faultlessly logical arrangement, its ease of acquirement, its flexibility, its facilities for natural growth, its smoothness of sound, and the fact that it has grown out of necessity, instead of being invented to satisfy an isolated theory.

First, as to simplicity and logical arrangement. The whole structure of the language is inflectional, like the Latin ; but there is but one declension of nouns, but one conjugation of verbs.

Nouns are declined by placing after the root word in the form of a *suffix*, the vowels "a, e, i," in their order. The nominative case is the root word ; the genitive or possessive terminates in "a," the dative in "e," the accusative or objective in "i."

The verbs are conjugated by *prefixes*, in which the same vowels are employed in the same order, "a, e, i, o, u," to indicate the imperfect, perfect, pluperfect, future and past future tenses, in regular succession.

Having mastered these two facts, the backbone of Volapük grammar is broken. The remaining rules are that the plural is always formed in just one way, by adding "s" to the singular, and that the verb governs the objective case. That the regular succession of the vowels "a, e, i, o, u," dominates the whole structure of the language, is plain.

There are no exceptions in the grammar ; and after it is once mastered, the only difficulty remaining is the memorising of words, as in any other language. But even here the difficulty is so small as to vanish on inspection. The root words of Volapük are taken from English, French, Latin, German and other languages ; but two-thirds are English, the next in number French, while the German and other tongues are responsible for but a small number of roots.

The only stumbling block on the threshold for an American entirely ignorant of any language but his own, lies in the pronunciation, but even here the difficulty is small, and vanishes on trial. The spelling is absolutely phonetic, every letter has but one sound, and the only real trouble found will lie in the pronunciation of the three dotted vowels : "dotted a, dotted o and dotted u," which must be mastered for the purposes of conversation.

Such is a fairly full outline of the structure of the great modern international language, and the reader need only ask himself if any language on earth, other than Volapük, *could* be described in so few words.

This simplicity and predominance of English roots makes Volapük easy of acquirement to Americans. A single week devoted to the study will enable any person of ordinary ability to write a business letter, with the dictionary beside him, with no fears of slips in grammar, and the command of words soon becomes so great that a single month's practice in a conversation club would make almost any one a fluent speaker.

But the American reader may still ask, what is the use of learning it? Simply because with Volapük and your own tongue you can already travel in remote parts of Europe, with the certainty of finding friends, and, because, in ten years more, at the present rate of increase, Volapük and English will carry a man over the known world.

Even at present, such is the enthusiasm for Volapük among those who have studied it, that an American Volapükist can open correspondence with perfect strangers of almost any nationality, and thus obtain knowledge, which, otherwise, could not be gained save by going to the countries themselves and learning from the natives.

Names of people willing to "spodön volapük" or correspond in the new language, are published monthly in the "Volapükabled Zenodik," and copied into all the other papers of the same kind. A late number offers such correspondence from St. Petersburg, Beirout, Buda Pesth and other places in Europe; while Chinese, Japanese and Turks are alike open to the advantage of a language which will rid them of the necessity of mastering the intricacies of our terrible Anglo-Saxon speech which they dread so much.

For a young man in business, especially if in the importing trade, a knowledge of Volapük is sure to be useful and may be invaluable. Letters in Volapük are constantly coming into all the great houses in the city, and a Volapük clerk can command a fair salary *now*. In ten years more all clerks will have to learn it, and the distinction at present enjoyed by a Volapükist will have vanished.

*Nu binom tim lenadön Volapüki.*

FREDERICK WHITTAKER.

